# Income-related health inequalities associated with the coronavirus pandemic in South Africa: A decomposition analysis

**DOI:** 10.1186/s12939-020-01361-7

**Published:** 2021-01-07

**Authors:** Chijioke O. Nwosu, Adeola Oyenubi

**Affiliations:** 1 The Impact Centre, Human Sciences Research Council, Cape Town, South Africa; 2 School of Economics and Finance, University of the Witwatersrand, Pretoria, South Africa

**Keywords:** COVID-19, Income-related health inequality, Health, South Africa, Concentration index, Concentration curve, National Income Dynamics Study-Coronavirus Rapid Mobile Survey

## Abstract

**Background:**

The coronavirus disease 2019 (COVID-19) has resulted in an enormous dislocation of society especially in South Africa. The South African government has imposed a number of measures aimed at controlling the pandemic, chief being a nationwide lockdown. This has resulted in income loss for individuals and firms, with vulnerable populations (low earners, those in informal and precarious employment, etc.) more likely to be adversely affected through job losses and the resulting income loss. Income loss will likely result in reduced ability to access healthcare and a nutritious diet, thus adversely affecting health outcomes. Given the foregoing, we hypothesize that the economic dislocation caused by the coronavirus will disproportionately affect the health of the poor.

**Methods:**

Using the fifth wave of the National Income Dynamics Study (NIDS) dataset conducted in 2017 and the first wave of the NIDS-Coronavirus Rapid Mobile Survey (NIDS-CRAM) dataset conducted in May/June 2020, this paper estimated income-related health inequalities in South Africa before and during the COVID-19 pandemic. Health was a dichotomized self-assessed health measure, with fair and poor health categorized as “poor” health, while excellent, very good and good health were categorized as “better” health. Household per capita income was used as the ranking variable. Concentration curves and indices were used to depict the income-related health inequalities. Furthermore, we decomposed the COVID-19 era income-related health inequality in order to ascertain the significant predictors of such inequality.

**Results:**

The results indicate that poor health was pro-poor in the pre-COVID-19 and COVID-19 periods, with the latter six times the value of the former. Being African (relative to white), per capita household income and household experience of hunger significantly predicted income-related health inequalities in the COVID-19 era (contributing 130%, 46% and 9% respectively to the inequalities), while being in paid employment had a nontrivial but statistically insignificant contribution (13%) to health inequality.

**Conclusions:**

Given the significance and magnitude of race, hunger, income and employment in determining socioeconomic inequalities in poor health, addressing racial disparities and hunger, income inequality and unemployment will likely mitigate income-related health inequalities in South Africa during the COVID-19 pandemic.

## Introduction

The coronavirus disease 2019 (COVID-19) has devastated many health systems and the global economy with dire consequences for individual and household welfare. While the pandemic has adversely affected virtually everybody, such deleterious effects have not been uniform, with the possibility that certain sections of society are more likely to be affected than others [1]. It can be hypothesized that already vulnerable individuals such as those who have lost their jobs, individuals in precarious employment, those living in poor housing and neighbourhoods and the poor in general are more likely to bear the brunt of the pandemic than the relatively well-off. This is not surprising given that labour market disengagement and forced confinement through lockdowns are two avenues through which the pandemic has affected many populations [2, 3].

In response to the devastation caused by the pandemic on global value chains and movement restrictions (outright lockdowns in some instances), many firms have resorted to furloughs or outright retrenchment of staff. For instance, Forsythe et al. [4] report a 30% reduction in job vacancy rates (representing a fall in labour demand) in the early months of the pandemic in the US. Moreover, as reported by Montenovo et al. [5], the employment losses associated with COVID-19 in the US exceeded those of the 2001 recession and the Great Recession of 2007–2009. An obvious consequence of such labour market disengagement is loss of income. For instance, about 50% of survey participants in the Kilts Nielsen Consumer Panel surveys in the US reported income losses due to COVID-19 [6].

South Africa has been significantly affected by the COVID-19 pandemic, with the country implementing one of the strictest lockdowns globally. Having declared a State of National Disaster on March 15, the country went into a total lockdown on March 26 – designated Level 5 restrictions – with only essential travel and services allowed [7]. This was later reduced to level 4 (the second highest level of restrictions which also involved significant restrictions on movement and economic activities) between 1 and 31 May. Level 3 restrictions, which allowed for some non-essential economic activities, only commenced on 1 June, lasting until 17 August, with the current level 2 restrictions commencing on 18 August 2020 [8]. Thus, over the last few months since the coronavirus pandemic in South Africa, there has been a significant drop in economic activities.

According to a Statistics South Africa (Stats SA) survey, 85% of businesses reported below-than-normal turnover, with 46.4% indicating temporary closure or paused trading activity due to COVID-19, while 36.8% expected their workforce to shrink [3]. Another survey by Stats SA indicates that the adverse income effects of the pandemic operated through at least two avenues: outright cessation of income generation, and reduction in income [9]. The survey indicated that the percentage of respondents who reported receiving no income increased from 5.2% before the lockdown to 15.4% by the sixth week of the lockdown. Moreover, a quarter of those surveyed reported a decrease in income during the lockdown. Another survey indicated that about three million South Africans lost their jobs between February and April 2020, with the poor and vulnerable most affected [10].

Such income and job losses would no doubt adversely affect health outcomes. The negative health impact of the COVID-19-induced employment and job losses is likely to operate via channels like reduced ability to purchase nutritious diets, poorer access to quality health care and ability to afford other necessities like electricity and water. For instance, another recent survey of South Africans – the COVID-19 Democracy Survey – indicates that 34% of adult South Africans were going to bed hungry during the lockdown [11] – substantially higher than 11.3% of the population who were vulnerable to hunger in 2018 [12]. Moreover, those living under inhospitable housing conditions like shacks are likely to find the lockdown more unbearable, raising the possibility of worsening (psychosocial) health outcomes. Given existing deep socioeconomic inequalities in South Africa mostly due to the legacies of apartheid, it is not surprising to imagine that the health outcomes of the poor are more likely to significantly worsen relative to the well-off during this crisis. As noted in popular media, COVID-19 has brought the steep economic inequalities in South Africa into sharp focus [13].

Available data indicate that indeed, COVID-19 more than proportionately affected the health of the poor in South Africa. Apartheid resulted in spatial segregation mostly along racial lines, with many of the poorer non-white population confined to poorly developed and overcrowded neighbourhoods popularly known as townships. Twenty-six years after the official end of apartheid, such race-biased spatial segregation largely remains in place. For instance, in the Western Cape, the epicentre of the pandemic as at June (making up 53% of infections nationally as at 21 June 2020) [14], reports indicate that Khayelitsha (a township) accounted for over 11% of infections despite making up only 6.7% of the provincial population. On the contrary, Stellenbosch (a more affluent and mostly white city) which constitutes about 2.7% of the provincial population only accounted for 1.5% of infections^1^ [15–17].

Such uneven impact of the pandemic is not only true for South Africa nor for COVID-19. Evidence from the US and elsewhere indicate more pronounced deleterious labour market effects of COVID-19 among workers in occupations that require face-to-face interactions as well as those in non-essential occupations, while the health impacts are more severe among males, older people and those with underlying health conditions [5]. Moreover, the jobs associated with higher levels of employment stability during the early days of the pandemic are generally associated with higher income [5]. Similarly, previous pandemics such as the Ebola Virus Disease demonstrated uneven adverse labour market effects especially in terms of sector and geography [18].

Given the foregoing, this paper ascertains the magnitude of income-related health inequality associated with the COVID-19 pandemic in South Africa. To achieve this, we compare income-related health inequality before the pandemic and during the pandemic-induced lockdown using panel data that links individuals over the two periods. We hypothesize that poor health was disproportionately concentrated on the poor and that the magnitude of the inequality in the COVID-19 era exceeded that of the pre-COVID-19 era. Furthermore, we decompose the COVID-19 era inequality to ascertain the factors that significantly determine such inequality. This will help in proposing key policy levers in order to mitigate income-related health inequalities in South Africa.

### Methods

### Data and key variables

Data were obtained from the last wave of the National Income Dynamics Study (NIDS) and the first wave of the NIDS-Coronavirus Rapid Mobile Survey (NIDS-CRAM). The only nationally representative panel dataset of South African residents, NIDS was collected biennially, with the first wave conducted in 2008 and the last wave (i.e. wave 5) collected in 2017. Two-stage stratified cluster sampling was used in the sampling design. In the original sample, about 400 primary sampling units (PSUs) were selected from 53 district council strata contained in Statistics South Africa’s 2003 master sample in the first stage. Thereafter, households were randomly sampled within each PSU while individuals were interviewed from within selected households [19].

NIDS-CRAM is a nationally representative survey that initially targeted more than 17 000 adults (with about 7 000 successful interviews conducted) based on the wave 5 adult sample of NIDS. It is a high frequency dataset to be collected monthly as a series of panel phone surveys between May and October 2020. The survey covers income and employment, household welfare, grant receipt and knowledge and behavior related to COVID-19. Stratified sampling with batch sampling was used as the sampling methodology. In this context, batch sampling means that sampled individuals were sent to fieldwork teams in batches of 2500 respondents, with individuals drawn randomly from 99 strata defined by a combination of rural/urban location, race, age and household per capita income decile. This sampling methodology allowed for flexibility to adjust the sampling rate per stratum as more information became available over the course of the fieldwork [20].

It must be stressed that because of a sample top-up done in wave 5 of NIDS due to non-random attrition (resulting in a top-up of the white, Indian and high-income population) [21] and the fact that NIDS-CRAM was based on the NIDS wave 5 sample, a suitable comparison would be between NIDS wave 5 (not earlier waves of NIDS) and NIDS-CRAM datasets [22]. This paper will therefore make use of the wave 1 version of the NIDS-CRAM survey conducted in May/June 2020 (coinciding with levels 4 and 3 lockdown) and the adult sub-sample of NIDS wave 5.

The outcome variable is self-assessed health (SAH). In each of these surveys, respondents were asked to describe their current health status. The responses were captured on a Likert scale comprising excellent, very good, good, fair and poor. We dichotomized each variable, with excellent, very good and good comprising one category, and fair and poor health status making up the other category. For ease of reference, we refer to these two groups as the better health and poor health categories respectively. A similar dichotomization of the five-category SAH variable has been implemented in prior studies [23]. Household income per capita (i.e. household income divided by household size) was used as an indicator of socioeconomic status against which health inequality was measured. Though consumption expenditure has been preferred to income as a measure of socioeconomic status in some developing country studies, we could not use consumption expenditure in this study given its unavailability in the NIDS-CRAM survey.

NIDS-CRAM comprised 7 074 observations. However, in order to enhance comparability between the NIDS wave 5 and NIDS-CRAM samples, we restricted the analysis to individuals who had non-missing observations for the variables used in the analysis in both waves (see Table 1). This resulted in an estimation sample of 4 124 observations. We ascertained whether key characteristics like gender, age, race and health status were significantly associated with inclusion in the estimation sample by regressing these variables on a sample inclusion/exclusion dummy variable. All covariates except age were not statistically significant at the 10% level (result available on request). But as shown in the analysis below, we also controlled for these variables in the main analysis.

It is important to highlight the differences in the manner in which otherwise similar variables were measured in NIDS and NIDS-CRAM. One, household income in NIDS was either based on aggregating the various income sources accruable to all income-receiving household members or by using a one-shot total household income provided by the oldest woman or a household member knowledgeable about the household’s living and spending patterns (for households where individual incomes were not available) [21]. Thus, to the extent that such income reports are correct, the resulting household income can be argued to be accurate. However, given that NIDS-CRAM was a telephonic survey on a random sample of NIDS wave 5, the household income question was a one-shot question that was asked of each respondent. A potential problem is that some respondents may not know what every household member earns. This is also a potential problem with NIDS wave 5, admittedly on a lower scale. This is because, while a majority of the household income variable in NIDS wave 5 was derived from aggregating the incomes of individual household members, a one-shot income variable obtained from a representative household member (similar to the approach adopted in NIDS-CRAM) was used to populate the household incomes of about 13% of households where such aggregation could not be carried out [21]. But as we subsequently show, the broad conclusions of this paper remain unchanged even when we use the full spectrum of the one-shot income question in NIDS wave 5 as a measure of 2017 household income. Moreover, we do not expect any bias in household income in NIDS-CRAM arising from the possibility that the respondent may not be knowledgeable about household income to be systematic across the distribution of household incomes given the randomness in the selection of respondents in the NIDS-CRAM survey.

Furthermore, given the fact that household per capita income was used for estimating the inequality measures, household size played an important role in the analysis. In NIDS, household size was obtained by aggregating all household members captured in a household roster. Expectedly in NIDS-CRAM, household size was obtained from a one-shot question to the respondent. While the former is preferable, we have no reason to doubt that most, if not all adults would be aware of the number of people living in their households at each point in time (especially given that this period coincided with the severe lockdown periods). Even when accurately reporting such a number might pose a challenge, the randomness of the sample persuades us that no systematic bias would likely result from deflating the household income with household size obtained in this manner.

Moreover, we believe that the use of income ranks, not actual income, in computing concentration indices (see Eq. (1) below) mitigates any bias that may arise from any possible misreporting of income in NIDS-CRAM especially given no evidence of systematic misreporting. To empirically test this, we estimated the Spearman correlation coefficient between the per capita household income ranks (in both data waves) of those who reported not losing their main source of income during the COVID-19-induced lockdown. The correlation coefficient: 0.6, was statistically significant (p < 0.01), implying that income ranks across the two waves were not independent for this subset of the population^2^.

### Analytical methods

#### Concentration curves

Income-related health inequality was depicted using concentration curves. A concentration curve depicts the cumulative share of the population who self-reported being in poor health against the cumulative population shares, ranked by household income per capita. A 45-degree line depicts the line of equality. If the concentration curve coincides with this line, it indicates that poor health is equally distributed across the income distribution, implying a proportional distribution. However, if poor health is more than proportionately concentrated on the poor (rich), the concentration curve would lie above (below) the 45-degree line [24].

While the concentration curve is important in depicting income-related inequality at each point in the income distribution for a health outcome of interest, it cannot be used to quantify the magnitude of such income-related inequality [25, 26]. Moreover, where concentration curves cross each other, it is not possible to determine dominance. For these reasons, it is therefore important to quantify the magnitude of income-related inequality in the health outcome of interest with a summary index; this necessitates the estimation of the concentration index.

#### Concentration indices

Given the foregoing, we also estimated concentration indices as an alternative measure of income-related health inequalities. The concentration index was computed as follows [24]:

1 $${C}_{S}=\frac{2}{{\mu }_{S}}cov\left(S,r\right)$$

where ${C}_{S}$ refers to the concentration index of SAH (*S*); ${\mu }_{S}$ refers to the mean of SAH, and *r* is the fractional rank of the individual/household in the income distribution. Thus, the concentration index is hereby defined as twice the covariance of the health outcome and the fractional rank of the individual in the income distribution divided by the mean of the health outcome.

Typically (i.e. for ratio-scale variables), the concentration index lies between the [-1,+1] interval. A negative (positive) index indicates a pro-poor (pro-rich) distribution of poor health, analogous to the concentration curve lying above (below) the line of equality, while a zero concentration index denotes a proportional distribution of poor health across income classes, similar to the concentration curve coinciding with the line of equality [24]. As noted elsewhere [24], a concentration index cannot be directly computed for a categorical variable like the original five-category SAH outcome in this paper. Even a dichotomization, as done here, does not solve the problem, as the bounds of the resulting concentration index are not − 1 and + 1, with the concentration index dependent on the mean of the health outcome. In this case, the lower and upper bounds of the concentration index become ${\mu }_{S}-1$ and ${1-\mu }_{S}$ respectively for large samples, with the implication that the feasible interval of the concentration index shrinks as the mean of the health outcome rises [27].

Given the foregoing, Wagstaff [27] suggested normalizing the concentration index by dividing through by ${1-\mu }_{S}$. However, Erreygers [28, 29] noted that such normalization is ad-hoc, proposing a more general normalization for ordinal outcomes, including dichotomous variables. Indeed, Wagstaff [30] has shown that the Erreygers [28] normalization (${E}_{S}$) is equivalent to:

2 $${E}_{S}=4\left(\frac{{\mu }_{S}}{b-a}\right){C}_{S}$$

where $a$ and $b$ are the lower and upper limits of the ordinal health indicator respectively; and ${\mu }_{S}$ and ${C}_{S}$ remain as earlier defined.

Decomposing income-related inequalities in poor health.

We decomposed the income-related inequalities in poor health using the Wagstaff et al. [31] approach. Thus, we specified a linear probability model of poor health as follows:

3 $${S}_{i}=\alpha +\sum _{k}{\beta }_{k}{z}_{ki}+{\epsilon }_{i}$$

where $\alpha$ and $\beta$ are parameters, and $\epsilon$ is the error term. Eq. (3) was appropriately weighted to the population while correcting for heteroscedasticity. We decomposed the concentration index in Eq. (1) as follows:

4 $${C}_{S}=\sum _{k=1}^{K}\left(\frac{{\beta }_{k}{\stackrel{-}{z}}_{k}}{{\mu }_{S}}\right){C}_{k}+\left(\frac{G{C}_{\epsilon }}{{\mu }_{S}}\right)$$

where $\left(\frac{{\beta }_{k}{\stackrel{-}{z}}_{k}}{{\mu }_{S}}={\eta }_{k}\right)$ denotes the elasticity of poor health to marginal changes in the *k*-th explanatory variable, while ${C}_{k}$ denotes the concentration index of the *k*-th explanatory variable. $G{C}_{\epsilon }$ refers to the generalised concentration index of the error term, and $\left(\frac{G{C}_{\epsilon }}{{\mu }_{S}}\right)$ represents the unexplained component. Given the lack of analytical standard errors for the estimation of Eq. (4), we used the jackknife replication method to estimate the standard errors while accounting for the sampling design of the NIDS-CRAM dataset [32].

The jackknife approach works by removing a PSU from a stratum one at a time so that the number of replications, $R,$is the number of PSUs in the data. Let $h=1,\dots ..L$ be the stratum index and $i=1,\dots ..{n}_{h}$ be the PSU index within a stratum. Then $R={n}_{1}+{n}_{2}\dots \dots \dots +{n}_{L}$, where ${n}_{h}$ is the number of PSUs in stratum $h$. If PSU $k$ in stratum $g$ is removed in the ${r}^{th}$ replicate, the replicate weights are defined by

$${w}_{{h}_{ij}}^{\left(gk\right)}=\left\{\begin{array}{*{20}c}0, h=g,i=k\\ \frac{{n}_{g}}{{n}_{g}-1}{w}_{{h}_{ij}}, h=g,i\ne k\\ {w}_{{h}_{ij}} h\ne g\end{array}\right.$$

where ${w}_{{h}_{ij}}$ and ${w}_{{h}_{ij}}^{\left(r\right)}$ represent the sampling weight of unit ${h}_{ij}$ and replicate weight of ${h}_{ij}$ in the ${r}^{th}$ replicate, where $r=gk$. The jackknife variance estimator is then defined by

$${v}_{J}=\sum _{h}\frac{{n}_{h}-1}{{n}_{h}}\sum _{i}\left\{{\widehat{\theta }}^{\left(hi\right)}-{\widehat{\theta }}^{h}\right\}$$

where ${\widehat{\theta }}^{\left(hi\right)}$ is the estimate with unit $i$ in statum $h$ removed from the dataset (see Kolenikov [32] for further details). We used this approach to estimate the standard errors for the components of the decomposition in Eq. (4).

## Results

### Descriptive statistics

Table 1 presents the descriptive statistics. Apart from NIDS wave 5 per capita household income and health outcome (required to compute the 2017 concentration index), all the reported variables were NIDS-CRAM values given that the decomposition of the income-related health inequality was only carried out for the COVID-19 era concentration index.

**Table 1:** Descriptive statistics

Variable	Mean/Percentage
Poor health	26.7%
Poor health (year = 2017)	8.7%
Household per capita income	R2540.8^a^
Household per capita income (year = 2017)	R4733.8
Age	41.3 years
Years of education	11.1
Male	45.3%
**Race dummies**	
African	78.1%
Coloured	10.0%
Asian	2.5%
White	9.4%
Employed and earning income	43.8%
**Dwelling type dummies**	
Formal dwelling	77.9%
Traditional dwelling (e.g. huts)	8.5%
Informal dwelling (e.g. shacks)	13.6%
Has chronic condition	19.9%
Household experienced hunger	23.4%
Has breathing problem	3.6%
Has fever, sore throat or cough	10.5%
**Number of observations**	**4 124**

NIDS wave 5 estimates weighted by wave 5 post-stratification weights; NIDS-CRAM estimates weighted by NIDS-CRAM design weights; Where year is not indicated, variables refer to the NIDS-CRAM survey (2020); ^a^South African Rands (US$1 = R17 (https://www.resbank.co.za/Research/Rates/Pages/SelectedHistoricalExchangeAndInterestRates.aspx)

Table 1 indicates a substantial increase (18 percentage points) in the prevalence of poor health between 2017 and the COVID-19 era. Moreover, while bearing in mind the difficulties inherent in comparing per capita household income over the two periods, nominal per capita household income declined by 46% over time. The average age of the population was 41 years (ranging from 18 to 102 years), while males comprised 45% of the population. Most of the population (78%) were Africans while those employed and earning income made up 44% of the population (in figures not reported, those employed but earning no income – probably furloughed workers – accounted for 3% of the population). Most of the population lived in formal housing structures while 14% lived in informal dwellings (such as shacks). Twenty percent of the population had chronic health conditions while 23% belonged to households where someone experienced hunger due to lack of food. In terms of symptoms similar to those of COVID-19, while 4% experienced breathing problems, 11% experienced fever, sore throat or cough.

Table 2 depicts the proportion of poor health across income quintiles in 2017 and the COVID-19 era.

**Table 2:** Prevalence of poor health by quintiles of per capita household income (%)

Quintiles	NIDS wave 5 (2017)	NIDS-CRAM (2020)
1	8.4	33.3
2	8.5	28.9
3	11.6	29.3
4	10.8	24.8
5	5.8	20.1
**Population**	**8.7**	**26.7**

NIDS wave 5 estimates weighted by wave 5 post-stratification weights; NIDS-CRAM estimates weighted by NIDS-CRAM design weights; Estimation sample = 4 124

Table 2 indicates that for the NIDS-CRAM population, the prevalence of poor health generally declined for higher income quintiles. For NIDS wave 5, while the richest quintile had the lowest prevalence of poor health, the negative relationship was not as pronounced as that of the NIDS-CRAM data. From the foregoing, we expect to find stronger evidence of pro-poor health inequalities in the COVID-19 era relative to 2017.

Pre-COVID-19 and COVID-19 era concentration curves.

**Fig. 1:**
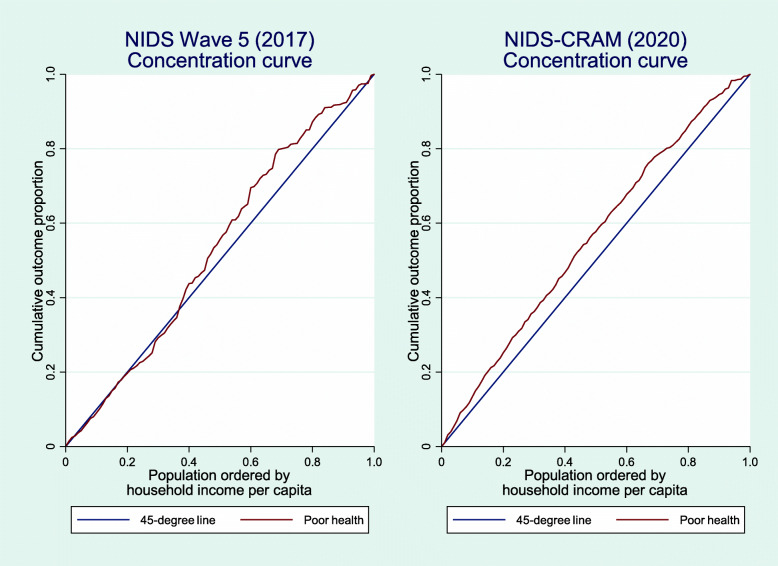
Concentration curves for poor health (2017 and 2020)

Figure 1 presents concentration curves for the pre-COVID-19 and COVID-19 periods.

As shown in Fig. 1, income-related health inequalities were generally concentrated on the poor given that both concentration curves largely lay above the 45-degree line. Moreover, we suspect that the COVID-19 era concentration index would be more pro-poor than the 2017 index given that the former generally lay everywhere above the line of equality while the latter curve mostly coincided with the line of equality for most parts of the poorest 40th percentile.

Pre-COVID-19 and COVID-19 era concentration indices.

To more definitely ascertain the relative magnitudes of income-related health inequalities in the pre-COVID-19 and COVID-19 periods, Table 3 reports the Erreygers-normalized concentration indices.

**Table 3:** Erreygers-corrected concentration indices for poor health (2017 and 2020)

Group	Period	
	NIDS Wave 5 (2017)	NIDS-CRAM (2020)
Female	0.013 (0.021)	-0.151*** (0.029)
Male	-0.042* (0.023)	-0.088** (0.044)
**Population**	**-0.022 (0.015)**	-0.123*** (0.026)

NIDS wave 5 estimates weighted by wave 5 post-stratification weights; NIDS-CRAM estimates weighted by NIDS-CRAM design weights; Estimation sample = 4 124; Standard errors in parentheses; *** *p* < 0.01, ** *p* < 0.05, * *p* < 0.1

The pro-poor population estimates for poor health in Table 3 for both the pre-COVID-19 and COVID-19 periods are in conformity with the graphical results in Fig. 1. The poor health concentration indices in 2017 and 2020 were − 0.022 and − 0.123 respectively. This indicates that the COVID-19 era concentration index was about six times that of the pre-COVID-19 index^3^. Furthermore, Table 3 indicates that pro-poor income-related inequalities in poor health were more pronounced among men relative to women in the pre-COVID-19 era while the converse obtained in the COVID-19 era^4^

Determinants of income-related inequalities in poor health in the COVID-19 period: A decomposition analysis.

Table 4 presents the results of the decomposition of the income-related health inequalities in the COVID-19 era.

**Table 4:** Determinants of income-related health inequalities in the COVID-19 era

	CI^a^ ($C_{k}$)	Elasticity ($\eta_{k}$)	Contribution ($\eta_{k} C_{k}$)	Contribution (%)
**Continuous covariates**				
Age in years	0.023***	0.212*	0.005	-4.08
	(0.003)	(0.115)	(0.003)	
Years of schooling	0.057***	-0.033	-0.002	1.63
	(0.005)	(0.129)	(0.007)	
Log of per capita household income	0.202***	-0.277**	-0.056**	45.67
	(0.007)	(0.119)	(0.024)	
**Qualitative covariates**				
Male (reference = female)	0.146***	0.015	0.002	-1.63
	(0.030)	(0.037)	(0.005)	
**Race (reference = White)**				
African	-0.286***	0.558***	-0.159***	129.67
	(0.034)	(0.107)	(0.032)	
Coloured	0.028*	0.015	< 0.001	-0.08
	(0.017)	(0.016)	(0.001)	
Asian	0.022*	0.007	< 0.001	-0.08
	(0.013)	(0.006)	(0.000)	
Employed and earning income (reference = not employed/not earning income)	0.441***	-0.037	-0.016	13.05
	(0.026)	(0.037)	(0.016)	
**Dwelling type (reference = formal dwelling)**				
Traditional dwelling (e.g. hut)	-0.078***	-0.007	0.001	-0.82
	(0.014)	(0.011)	(0.001)	
Informal dwelling (e.g. shack)	-0.075***	-0.009	0.001	-0.82
	(0.020)	(0.018)	(0.001)	
Chronic illness (reference = no chronic illness)	-0.021	0.053**	-0.001	0.82
	(0.022)	(0.021)	(0.001)	
Household experienced hunger (reference = no household experience of hunger)	-0.217***	0.051**	-0.011**	8.97
	(0.021)	(0.024)	(0.005)	
Has breathing problem (reference = has no breathing problem)	0.005	0.031**	< 0.001	-0.08
	(0.014)	(0.013)	(0.000)	
Has fever, sore throat or cough (reference = has none of these symptoms)	0.03	0.037**	0.001	-0.82
	(0.019)	(0.017)	(0.001)	
Error			0.236***	
			(0.035)	

^a^Concentration index; Estimates weighted by NIDS-CRAM design weights; Estimation sample = 4 124; Jackknife standard errors with 1 014 replications in parentheses; *** *p* < 0.01, ** *p* < 0.05, * *p* < 0.1

Table 4 indicates that race (being African compared to white), per capita household income and household hunger significantly contributed to income-related inequalities in poor health, accounting for 130%, 46% and 9% of the estimated income-related inequality. Moreover, each of them had a pro-poor effect on health inequalities, implying that they contributed to worsening the burden of poor health on the poor in South Africa. Also, while not being statistically significant, income-earning employment accounted for 13% of the total concentration index. In addition, while some variables did not significantly/substantially determine health inequalities, Table 4 indicates that they had a statistically significant relationship with health (via their elasticities). For instance, age, having a chronic health problem and exhibiting symptoms similar to COVID-19 (breathing problem, fever, sore throat or cough) were all positively and significantly associated with poor health. Moreover, being male, age and having more years of schooling were expectedly pro-rich, while living in traditional and informal dwelling were both pro-poor.

The pro-poor effect of being African (relative to white) on inequality implies that eliminating/mitigating the positive relationship between being African and being in poor health (i.e. the positive elasticity) and/or the concentration of Africans (relative to whites) among the poor (i.e. the negative African concentration index) will reduce the extent to which poor health is disproportionately borne by the poor relative to what currently obtains. The same applies to household hunger, while mitigating income inequality and providing paid employment to those willing and able to work will achieve a similar outcome.

## Discussion

This paper has tested the central hypothesis that the COVID-19 pandemic in South Africa is associated with more deleterious health effects on the poor relative to the well-off. We contended that given the enormous disruption caused by the pandemic and the associated nationwide lockdown as well as the credible possibility that its effects (such as via the labour market, accentuated historical racial inequalities and overall living standards) will disproportionately disadvantage the poor, income-related health inequalities would become more pro-poor in the COVID-19 era than in the pre-COVID-19 era. As indicated above, this is the case, with the magnitude of income-related health inequality in the COVID-19 era six times what obtained in 2017.

Moreover, we found that income-related health inequality was higher among women than among men in the COVID-19 period. We suspect that this may not be unconnected with the fact that women have been more adversely affected by COVID-19-related lockdowns and economic disruption. For instance, women were more likely to lose their jobs, be burdened with additional childcare responsibilities and suffer from gender-based wage disparities due to the pandemic in South Africa [33]. This has the potential to further exacerbate the socioeconomic disparities between majority of women and the relatively few women who are economically secure.

The decomposition results highlight race, income and hunger as the significant contributors to income-related health inequalities in the COVID-19 era. Moreover, while not being statistically significant, income-earning employment also had a nontrivial contribution to increased health inequality.

The finding that race mediates the impact of COVID-19 on welfare corroborates prior evidence for South Africa. It has been noted that blacks/Africans are among the worst affected by the COVID-19 pandemic in South Africa [34]. One of the avenues through which such steeper African racial gradient occurs is higher exposure to hazardous jobs (by working as cleaners, nurses and in fumigation of contaminated areas). Indeed, the relative disadvantage of historically disadvantaged racial groups to pandemics is well known especially in the present situation. For instance, African Americans have disproportionately high infection and mortality rates due to COVID-19 in the United States [35]. Moreover, the pro-poor African concentration index is not surprising given that Africans are over-represented among the poor in South Africa. For instance, the real annual mean household expenditure for households headed by whites was seven times that of households headed by Africans in 2015 (131 198 Rands i.e. US$7 718, and 18 291 Rands i.e. US$1 076 for whites and Africans respectively) [36]. In fact, using median household expenditure, racial inequality appears worse as the white median expenditure was eleven times that of Africans according to the same report.

Another way through which race (being African) predicts poor health in South Africa is through access to quality health care. The deep inequalities/inequities in the South African health system are well documented [37, 38]. The South African health system is highly segmented, with a private sector similar to developed world health systems while the severely under-resourced public sector is overburdened by serving majority of the population [37]. The well-resourced private sector is mainly financed via membership of medical aid schemes which are unaffordable to majority of the population (mostly Africans). Available data indicate that in 2018, only about 16% of South Africans were members of medical aid schemes, with only 10% of Africans belonging to such schemes compared to 73% of whites [12]. However, as reported by the World Health Organization^5^, private health expenditure accounted for about 44% of current health expenditure in 2017 (when only 17% of the population belonged to medical aid schemes). Given that Africans are less likely to belong to private medical aid schemes than other racial groups (especially whites) – thus, more likely to use the overburdened public health sector, it is not surprising that a positive relationship exists between poor health and race.

Hunger, which is an extreme form of food and nutrition insecurity, predisposes one to poor health outcomes. Therefore, it is not surprising that hunger was significantly associated with worsening income-related health inequality. Copious studies corroborate our findings of a positive relationship between hunger and poor health, as well as the fact that hunger is disproportionately borne by the poor [39, 40]. In particular, the fact that hunger is significantly pro-poor (p < 0.01) is worrying and indicates that the rights-based approach adopted by the South African constitution towards food and nutrition security, where the right to food is inextricably linked to the right to life and dignity (see Section 27 (1) (b) of the Constitution of the Republic of South Africa) is being undermined in the COVID-19 era [41]. This indicates that at the very least, various policies aimed at addressing food and nutrition insecurity in South Africa like the National Food and Nutrition Security Plan, the Agricultural Policy Action Plan, and the National Policy on Food and Nutrition Security are not sufficient for shielding the poor and vulnerable from hunger in the face of a pandemic of this magnitude.

Moreover, COVID-19 has exacerbated the threat of hunger especially among the poor. For instance, the lockdown necessitated the closure of schools, resulting in the cessation of the school feeding programme implemented under the National School Nutrition Programme. This programme serves as a major source of food for over nine million pupils and students mostly attending low income, no-fee paying (otherwise known as Quintile 1 – Quintile 3) schools [42]. This, and the massive loss of income generating opportunities due to job losses, is worrying and highlights the urgent need to avert a hunger crisis. For instance, it has been found that about three million jobs were lost between February and April 2020 (with April indicating the period of the hard lockdown) [10]. Fortunately, a court decision has recently mandated the provision of food to these learners irrespective of school closure [43]. One hopes that this will mitigate the pro-poorness of hunger and ultimately the contribution of hunger to income-related health inequality in the future.

In addition, the significant contribution of income to worsening health inequality conforms to the majority of available evidence on the impact of income inequality on health, with prior evidence suggesting a causal relationship [44]. One clear fact in South Africa is that the poorer and more vulnerable segments of society suffered more as a result of COVID-19 and the associated hard lockdown. For instance, a recent study found that the likelihood of low earners (earning below 3 000 Rands, i.e. US$176 per month) losing their jobs between February and April 2020 was about eight times that of high earners (earning more than 24 001 Rands, i.e. US$1 412 per month) [45]. Such a relatively high probability of job loss among already economically compromised individuals and households would not only exacerbate income inequality but is likely to contribute to worsening health outcomes among the poor given their further limited ability to meet basic needs like food and medication.

Furthermore, though income-earning employment was not statistically significant, it had a nontrivial contribution to health inequality (numerically higher than hunger). Thus, the combination of the fact that gainful employment is negatively associated with poor health and its concentration on the relatively well-off resulted in worsening the health disparities between the poor and the rich [46, 47]. Indeed, the pro-rich concentration index of employment supports the above finding of high earners being minimally impacted by job losses during the lockdown.

Implications for policy.

The central contention of this paper is that poor health is disproportionately borne by the poor in South Africa and that such income-related health inequalities appear to have become substantially more pronounced in the COVID-19 era relative to the pre-COVID-19 period. We believe that this outcome can at least be attributed to the disproportionate adverse impact of the pandemic and the associated lockdown on the poor especially by reinforcing historical racial and income inequalities and engendering a food crisis. Furthermore, massive job cuts (which disproportionally affected the already worse off) are likely to further burden the poor with health challenges. In this sense, such health inequalities in South Africa at least partly suggest the existence of health inequities, “i.e. health inequalities that are socially produced” [48].

To confront these challenges, bold actions are necessary to address historical racial inequalities in the country. First, the negative relationship between race (being African in particular) and poor health is a sad indictment of the country a quarter century since the end of apartheid. Given the deep racial inequalities and inequities in accessing quality health care, it is important to implement policies that will level the playing field in the provision of universal access to quality health care. In addition to addressing other root causes of race-related poverty, such measures must include the achievement of equity in health sector funding, where most of the available resources for the health sector are directed toward serving majority of the population. Perhaps, a well designed and implemented National Health Insurance Scheme will significantly mitigate these racial inequalities in health.

Furthermore, there is an urgent need to eliminate hunger as well as substantially mitigate all other forms of food and nutrition insecurity in South Africa. The above results indicate that not only is hunger positively related to poor health, poor people are more than proportionately likely to face hunger than the relatively well-off. It should not be the case that anybody should face hunger, especially in an upper middle income country like South Africa. So far, some short term policy options that are likely to mitigate the deleterious effect of hunger on health inequalities include the monthly COVID-19 Social Relief of Distress (SRD) grant of R350 (US$20.59) earmarked for unemployed South Africans with no alternative source of income (for six months), as well as the top up of the various grants that form part of South Africa’s basket of social assistance programmes^6^. While commendable, it is obvious that these social assistance packages are insufficient for addressing the hunger crisis during this period. Furthermore, the exclusion of non-refugee temporary residents from benefitting from the SRD grant will likely have negative consequences for health inequalities. Moreover, available evidence indicates gross inefficiencies and uncertainty in the disbursement of the SRD grant [49]. Therefore, in addition to improving the efficiency and effectiveness of existing relief measures, we suggest the expansion of the basket of zero-rated foodstuff to include more basic and essential foodstuff in the immediate period as a complementary policy to alleviate hunger in the country. In the medium-to-long term, employment and economic growth incentives should be considered as a means of improving overall incomes, especially for the poor and marginalized.

Finally, this paper reinforces the fact that high income inequality has far-reaching consequences for health. That South Africa is one of the most income unequal countries globally is no longer news. It is therefore imperative that the country speed up comprehensive reforms especially with regards to labour market access, welfare and access to quality health care.

The main strength of this paper is that it highlights the existence, and worsening of income-related health inequalities during the COVID-19 period relative to the pre-COVID-19 period. Thus, the paper contributes to the growing global evidence on health inequalities during the current pandemic [35, 50]. Such evidence is important for an early targeting of the key predictors of income-related health inequalities in order to mitigate the overall impact of the pandemic in South Africa.

However, one of the limitations of the study is the nature of the data used in the analysis. As earlier highlighted, the pre-COVID-19 data on income and household size appear to be more objective than their COVID-19 era counterparts due to the impossibility of conducting an in-person survey during a pandemic-induced lockdown. That said, we believe that the randomness of the sample mitigated any possible bias, while basing the analysis on the same individuals in both periods enhanced comparability. Moreover, one would have preferred the pre-COVID-19 data to have been collected immediately before the COVID-19 lockdown – say in February 2020, rather than 2017. Unfortunately, data from 2017 was the most recent available nationally representative survey for South Africa upon which the NIDS-CRAM sample was based. In addition, we were not able to include some potentially key predictors of health status like marital status and depression [51, 52] due to the non-inclusion of these variables in the NIDS-CRAM survey. These variables are likely to significantly predict overall health status, while their non-inclusion possibly contributed to the statistical significance of the error term in the decomposition results.

## Conclusions

Understanding the nature and key determinants of income-related health inequalities during the period of the COVID-19 pandemic is important for designing and implementing appropriate policies aimed at tackling health disparities. This study has ascertained that the poor bore a higher burden of ill health before and during the pandemic, with the problem exacerbating during the pandemic. Race, income and hunger were the significant contributors to such inequality, with employment also playing an important role. Therefore, addressing disparities associated with these factors – which constitute social determinants of health – will likely go a long way in protecting the health of the poor, thus mitigating the health disparities associated with income in South Africa.

## Data Availability

The datasets analysed during the current study are available in the Datafirst repository, https://www.datafirst.uct.ac.za/dataportal/index.php/catalog/central.

## Abbreviations

COVID-19: Coronavirus disease 2019 [pandemic]
NIDS: National Income Dynamic Study
NIDS-CRAM: NIDS-Coronavirus Rapid Mobile Survey
PSU: Primary Sampling Unit
SAH: Self-assessed Health
SRD: COVID-19 Social Relief of Distress grant
Stats SA: Statistics South Africa

## Footnotes

1 Population proportions are based on 2011 Census population figures.

2 The correlation coefficient using the one-shot income variable in 2017 was 0.5 (p < 0.01).

3 The results did not change when the aforementioned one-shot income question (which is similar to the NIDS-CRAM income variable) was used to estimate the 2017 concentration index. This yielded a pre-COVID-19 concentration index of -0.039 (p < 0.01), indicating that the COVID-19 era index was three times the value in 2017.

4 We re-estimated the concentration indices with the original five-category SAH variable, as well as grouping excellent-fair health together and poor health separately. The conclusions remained similar to what was reported here (results available on request), with the NIDS wave 5 overall and male concentration indices for the five-category classification being statistically significant at 10% and 5% respectively, while none of the NIDS wave 5 indices for the excellent – fair health vs. poor health classification was statistically significant at conventional levels

5 https://apps.who.int/gho/data/node.main.GHEDPVTDCHESHA2011?lang=en.

6 In his recent Medium Term Budget Policy Statement, the finance minister announced that the top-up grants will come to an end in November 2020, while the SRD grant will be extended to January 2021 (https://www.groundup.org.za/article/covid-19-grant-extended-january/).
